# A hidden ally in laparoscopic cholecystectomy: quadratus lumborum block and the quest for pain-free recovery

**DOI:** 10.3389/fsurg.2025.1710676

**Published:** 2025-10-23

**Authors:** Serge Chooklin, Serhii Chuklin

**Affiliations:** Surgical Center, Saint Paraskeva Medical Center, Lviv, Ukraine

**Keywords:** laparoscopic cholecystectomy, pain, analgesia, regional anesthesia, quadratus lumborum block

## Abstract

Laparoscopic cholecystectomy (LC) is a minimally invasive procedure; however, it is frequently associated with considerable postoperative pain, which in some cases may progress to chronic pain. The underlying mechanisms are multifactorial and include trocar insertion, wound trauma, surgical manipulation of the gallbladder and adjacent organs, carbon dioxide insufflation, diaphragmatic irritation with referred shoulder pain, local inflammation, and, occasionally, nerve injury (0.02%–1%). Effective pain management is crucial not only for patient comfort but also for minimizing postoperative complications and facilitating faster recovery. The quadratus lumborum block (QLB) is an ultrasound-guided regional anesthetic technique that has gained increasing recognition as a component of multimodal analgesia for LC. By depositing local anesthetic adjacent to the quadratus lumborum muscle, the QLB can interrupt thoracolumbar nerve transmission, thereby providing both somatic and visceral analgesia. Across randomized and prospective studies, QLB is often associated with lower early postoperative pain scores, delayed time to rescue analgesia, and reduced opioid use; however, several trials report no significant differences or equivalence vs. other regional techniques (e.g., TAPB, ESPB) within multimodal analgesia. These mixed results likely reflect heterogeneity in QLB approach (posterior, lateral, anterior), injectate volume/concentration, comparators, and outcome definitions. The extent of analgesic coverage depends on the type of QLB performed, the administered volume of anesthetic, and patient-specific anatomical variations. Accordingly, the objective of this narrative review is to synthesize adult clinical evidence on QLB for LC, compare approach-specific analgesic and opioid-sparing effectiveness with alternative regional techniques and standard care, evaluate safety. This review summarizes current evidence on the use of QLB in LC, with a focus on its mechanisms, techniques, clinical efficacy, and limitations. Although QLB appears promising as an effective opioid-sparing strategy, given the heterogeneity and risk-of-bias concerns across studies, conclusions are moderated, and high-quality, standardized RCTs are needed.

## 1 Introduction

Laparoscopic cholecystectomy (LC) is one of the most frequently performed procedures for gallstone disease. Accordingly, patient comfort and the overall perioperative experience are as important as surgical quality and clinical outcomes. Despite its minimally invasive nature, many patients experience severe abdominal and shoulder pain following surgery, often requiring strong postoperative analgesia (1). Pain is not only a determinant of patient well-being but also a critical factor influencing cardiovascular and pulmonary complications, as well as emotional recovery during the postoperative period. The principal contributors to pain after LC include pneumoperitoneum, surgical dissection, and incisions at trocar sites (2).

Effective postoperative analgesia enhances patient comfort and facilitates early mobilization. Given the typically short duration of hospital stay, strategies that optimize pain relief while minimizing postoperative nausea and vomiting are essential to enable timely discharge (3). Conversely, inadequate analgesia may delay wound healing, prolong exudation, increase the risk of thromboembolic and pulmonary complications, and predispose patients to chronic neuropathic pain (4).

Current approaches to pain management include systemic opioids, nonsteroidal anti-inflammatory drugs (NSAIDs), pregabalin, gabapentin, intraperitoneal local anesthetic instillation, epidural analgesia, and paravertebral blocks (5). However, opioid administration is associated with considerable adverse effects, such as nausea, vomiting, constipation, and respiratory depression (6). Reducing postoperative opioid use has therefore become a major priority in the context of the opioid crisis in high-income countries (7, 8). Although NSAIDs are effective, they carry a risk of gastrointestinal complications. Epidural analgesia provides robust pain control and supports pulmonary recovery but is associated with risks such as dural puncture, nerve injury, bleeding, infection, hypotension, bradycardia, and urinary retention (1).

The PROcedure-SPECIFIC Postoperative Pain Management (PROSPECT) group has recently updated its evidence-based recommendations for analgesia after LC (9). Current guidelines emphasize the use of multimodal basic analgesia, consisting of oral or intravenous paracetamol in combination with NSAIDs or COX-2 inhibitors, administered preoperatively or intraoperatively and continued postoperatively, unless contraindicated. Intravenous dexamethasone is recommended as an adjunct for both its analgesic and antiemetic properties. Regional techniques, such as port-site wound infiltration with long-acting local anesthetics or intraperitoneal instillation, are also advised. Transversus abdominis plane (TAP) and erector spinae plane (ESP) blocks are considered effective second-line options in selected patients, while opioids should be reserved strictly for rescue analgesia. Surgical strategies associated with improved postoperative pain outcomes include the use of low-pressure pneumoperitoneum (<12 mmHg), three-port rather than four-port laparoscopy, umbilical port extraction of the gallbladder, thorough aspiration of residual CO_2_, and intraoperative saline irrigation. Nevertheless, despite advances in anesthetic techniques, a significant proportion of patients continue to experience pain after LC (10).

Preventive multimodal analgesia is now recognized as the cornerstone of postoperative pain management, with regional anesthesia techniques playing a pivotal role (11, 12). These approaches not only reduce pain intensity but also minimize systemic analgesic requirements and their associated adverse effects, thereby improving patient satisfaction (1). The widespread availability of ultrasound guidance has further enhanced the safety and precision of these procedures. Peripheral nerve blocks, in particular, provide superior pain control compared with systemic NSAIDs or opioids. A detailed understanding of pain pathways and the anatomy of nociceptive transmission enables targeted interruption of pain signals at multiple levels (13). There is a growing emphasis on integrating regional anesthesia into multimodal regimens (14). Following LC, techniques used include paravertebral block (15), rectus sheath block (16), transversus abdominis plane block (17), intercostal nerve block (18), subcostal transversus abdominis plane block (19), thoracic epidural (20), erector spinae plane block (15), and quadratus lumborum block (QLB) (21).

Among regional techniques, the QLB has emerged as particularly valuable, providing effective somatic and visceral analgesia after LC (21). Beyond analgesia, QLB may blunt the perioperative inflammatory response and facilitate a faster return to baseline physiological function. However, head-to-head comparisons among regional techniques remain limited, leaving their relative efficacy in LC uncertain. Objective of this narrative review: to provide a structured synthesis of adult studies evaluating any QLB approach for LC; compare its analgesic and opioid-sparing effects with alternative regional techniques and standard care; summarize safety outcomes; and outline priorities for future randomized trials.

## 2 Methods

This article is a narrative review. We chose a narrative design—rather than a systematic, integrative, or scoping review—because the evidence on QLB for LC is highly heterogeneous in block technique (posterior, lateral, and anterior/intramuscular approaches; local-anesthetic doses and adjuvants), comparators (e.g., TAPB, ESPB, port infiltration, or placebo), perioperative co-analgesic protocols, and outcome definitions/timing. Under such heterogeneity, a formal meta-analysis would require strong—and potentially misleading—modeling assumptions; a scoping review would emphasize mapping over critical appraisal and typically omit risk-of-bias judgements; and an integrative review would broaden to qualitative or non-comparative evidence that does not directly answer our clinical question. A structured narrative synthesis allows us to integrate anatomical and technical context, evaluate the direction and consistency of effects, and explicitly qualify certainty where trials conflict.

We searched MEDLINE (PubMed), Embase, Scopus, and Google Scholar for studies published from 2015 through 2025 and supplemented this with backward citation screening of eligible papers and recent reviews; earlier foundational anatomy/technique articles (pre-2015) were consulted solely for mechanistic context. A representative search strategy combined controlled vocabulary and free-text terms, including “quadratus lumborum,” “QL block,” “laparoscopic cholecystectomy,” “analgesia,” and “pain.” We included randomized and non-randomized comparative studies evaluating any QLB approach in adult LC that reported at least one postoperative analgesic outcome (pain at rest or with movement at standardized intervals, time to first rescue, cumulative opioid consumption, or adverse events). We excluded case reports/series without a comparator, pediatric cohorts, open cholecystectomy, and studies in which the effect of the block could not be isolated from other interventions. Two reviewers independently screened titles/abstracts and full texts, resolved disagreements by consensus, and extracted study design, setting, sample size, QLB technique, comparator, anesthetic and co-analgesic regimens, outcome definitions and time points, and safety events. Risk of bias was assessed at the domain level using the Cochrane Risk of Bias 2 (RoB 2) tool for randomized trials and ROBINS-I for non-randomized comparative studies.

## 3 Mechanism of pain after laparoscopic cholecystectomy

Compared with conventional open surgery, LC is generally associated with less postoperative pain owing to its minimally invasive nature (22). Nevertheless, patients frequently report discomfort that arises from multiple sources, which can be broadly categorized into parietal, visceral, and referred pain (23, 24).

- Visceral pain is primarily related to gallbladder excision and irritation of the parietal peritoneum and diaphragm caused by residual carbon dioxide retained or dissolved within the abdominal cavity.
- Parietal pain results from trocar penetration through the abdominal wall and from thermal injury induced by electrocoagulation.
- Referred pain, typically localized to the shoulder, is attributed to diaphragmatic irritation transmitted *via* the phrenic nerve.

Additional factors include bile spillage and local inflammatory responses within the gallbladder bed, liver, diaphragm, and parietal peritoneum, all of which exacerbate pain and contribute to postoperative nausea (24).

Incisional pain may further compromise respiratory physiology by encouraging shallow breathing and restrictive ventilatory patterns, thereby predisposing patients to hypoxemia and pulmonary complications. The severity of postoperative pain thus becomes a decisive factor in determining the trajectory of recovery. Tissue trauma, organ manipulation, and pneumoperitoneum activate inflammatory cascades that not only intensify acute discomfort but may also prolong convalescence and impair functional rehabilitation (24). Collectively, these mechanisms explain why some patients develop severe acute pain after LC and why inadequate management can predispose individuals to persistent or chronic pain syndromes (25).

## 4 Types of quadratus lumborum blocks

**Figure 1:**
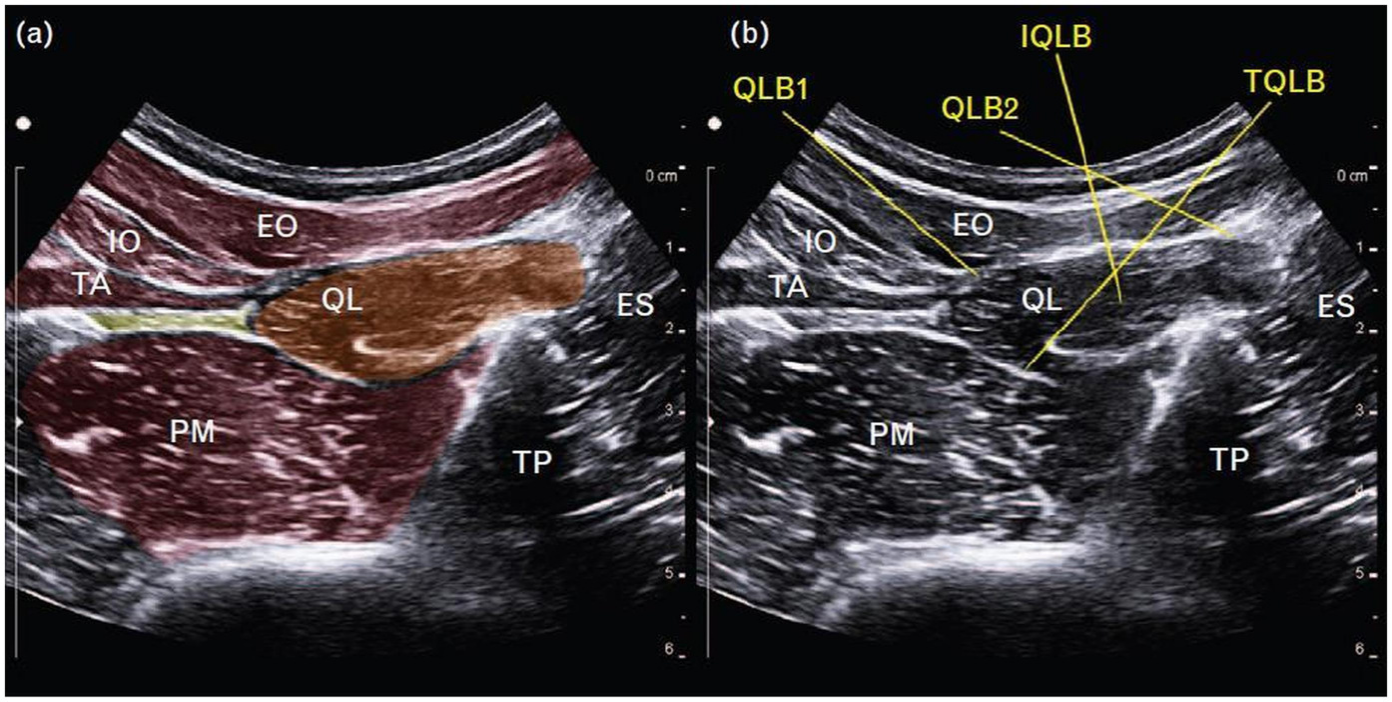
**(a)** Schematic representation of the quadratus lumborum region on ultrasound. **(b)** Ultrasound images demonstrating four different quadratus lumborum block approaches. EO, external oblique; ES, erector spinae; IO, internal oblique; IQLB, intramuscular quadratus lumborum block; QL, quadratus lumborum; QLB1, quadratus lumborum block 1; QLB2, quadratus lumborum block 2; TA, transversus abdominis; TP, transverse process; TQLB, transmuscular quadratus lumborum block [Reproduced from Korgvee et al. (29). © Wolters Kluwer Health, Inc. Published with permission under STM Permissions Guidelines].

The QLB is an ultrasound-guided fascial plane technique in which local anesthetic is deposited adjacent to the QL muscle to target thoracolumbar nerves (26). The method was first described by Blanco in 2007 (27) and has since gained widespread application across multiple surgical specialties. Several approaches have been developed, each distinguished by the position of the needle relative to the QL muscle and its surrounding fascial layers (Figure 1) (28, 29). A thorough understanding of the thoracolumbar fascia (TLF) is essential for understanding the mechanisms underlying these variants (30).

The TLF is a multilayered structure extending from the thoracic to the lumbar region. It encloses the paraspinal muscles and provides potential channels for cranio-caudal spread of local anesthetic (31). Three layers are traditionally identified: the anterior layer lies anterior to the QL, the middle layer separates the QL from the erector spinae (ES), and the posterior layer envelops the ES. Medially, the anterior layer fuses with the fascia of the psoas major (PM), whereas laterally it continues as the transversalis fascia. Anesthetic deposited between the anterior TLF and the QL can extend cranially beneath the arcuate ligament into the endothoracic fascia, with the potential to reach the thoracic paravertebral space (32).

Among the fascial structures, the lumbar interfascial triangle (LIFT) is of particular importance. It is formed by the junction of the middle lumbar fascia with the deep lamina of the posterior layer at the lateral margin of the ES. This anatomical site provides a favorable compartment for anesthetic deposition during posterior QLB (33). Furthermore, the dense network of sympathetic fibers and mechanoreceptors embedded within the TLF is thought to augment the analgesic effect of the block.

**Figure 2:**
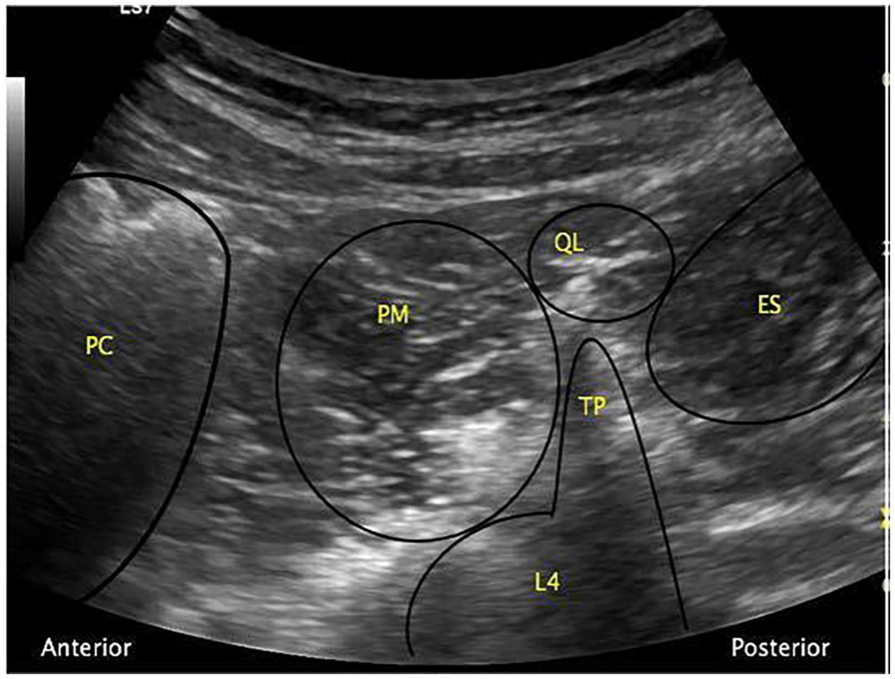
The “shamrock sign” obtained with a curvilinear ultrasound probe. ES, erector spinae muscle; L4, fourth lumbar vertebra; PC, peritoneal cavity; PM, psoas muscle; QL, quadratus lumborum muscle; TP, transverse process [Reproduced from Nee and McDonnell (35). © 2025 World Federation of Societies of Anaesthesiologists. Distributed under the Creative Commons Attribution License, CC BY 4.0].

In clinical practice, a curvilinear ultrasound probe (2–6 MHz) is most frequently used; however, in thinner patients, a linear probe may be sufficient. When the probe is positioned transversely above the iliac crest, the characteristic “shamrock sign” becomes visible, serving as a key sonographic landmark (Figure 2) (34–36).

Currently, four principal variants of QLB are described, although nomenclature varies among authors (37):

- QLB type 1 (lateral): injection at the anterolateral border of the QL;
- QLB type 2 (posterior): injection at the posterolateral surface of the QL;
- QLB type 3 (transmuscular, TQL, or anterior): injection between the QL and PM;
- QLB type 4 (intramuscular): injection directly into the QL muscle.

Another classification categorizes these techniques according to the trajectory of the needle tip relative to the QL muscle, distinguishing lateral, posterior, and anterior approaches (37, 38). Each technique differs in its anatomical target and pattern of spread, thereby determining the extent of dermatomal coverage, degree of sympathetic involvement, and duration of analgesic effect.

### 4.1 Lateral quadratus lumborum block

The type 1 QLB (QLB1)—also referred to as the lateral QLB—is performed by depositing local anesthetic deep to the aponeurosis of the transversus abdominis muscle (39). Because the injection site lies lateral to the QL muscle, at its interface with the transversalis fascia, this technique is frequently regarded as a fascial plane block directed primarily at the transversalis fascia.

QLB1 shares certain similarities with the posterior TAPB, in which anesthetic is placed between the internal oblique and transversus abdominis muscles of the posterolateral abdominal wall. The key distinction is that in the TAPB, the injectate remains in a more superficial plane, whereas in QLB1 it is delivered into the deeper fascial compartment. A notable advantage of QLB1 is the potential for cranial spread of anesthetic into the thoracic paravertebral space, thereby extending the range of analgesia (40).

**Figure 3:**
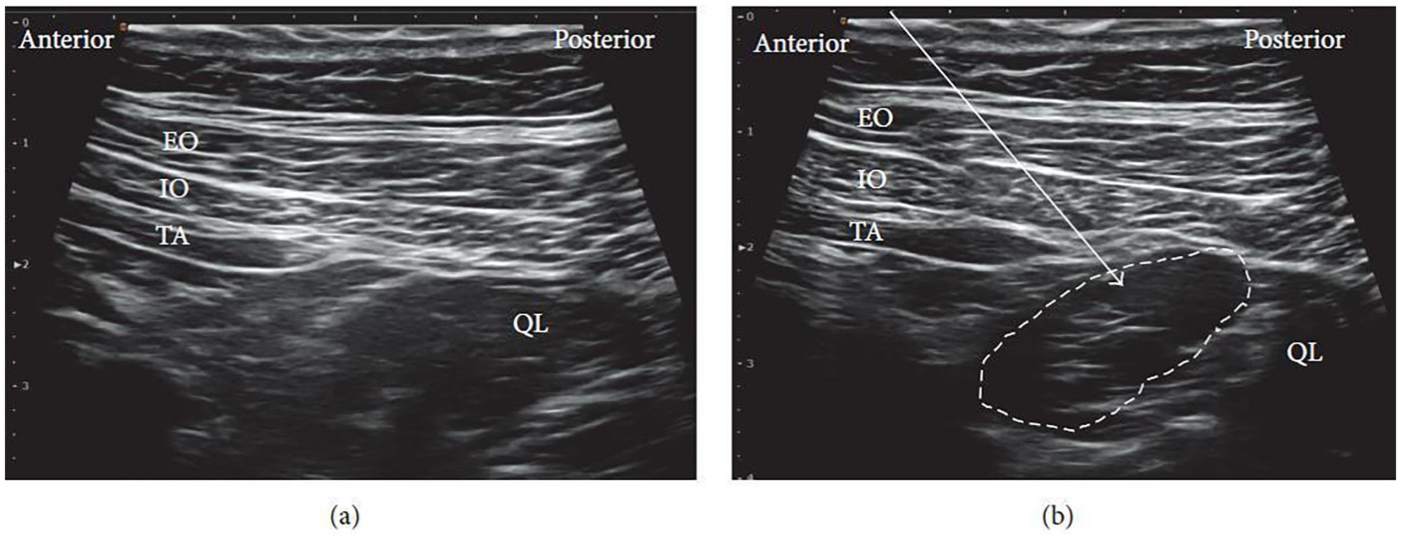
Ultrasound images of lateral QLB: **(a)** before injection and **(b)** after injection. EO, external oblique; IO, internal oblique; TA, transversus abdominis; QL, quadratus lumborum. White arrow: needle trajectory; white dotted line: spread of local anesthetic [Reproduced from Ueshima et al. (28). © John Wiley & Sons, Inc. Distributed under the Creative Commons Attribution License, CC BY 4.0].

For the procedure, the patient is typically positioned supine. A high-frequency linear ultrasound probe is applied over the Petit triangle until the QL muscle is clearly visualized (Figure 3a). The needle is advanced toward the anterolateral surface of the QL at the junction with the transversalis fascia, and local anesthetic is injected at this site. Proper placement of the injectate beneath the transversus abdominis aponeurosis is confirmed using real-time ultrasound visualization (Figure 3b).

### 4.2 Posterior quadratus lumborum block

The type 2 QLB (QLB2), also known as the posterior QLB, is performed by depositing local anesthetic posterior to the QL muscle. Compared with the lateral and anterior approaches, this technique offers several advantages: the injection site is more superficial, ultrasound visualization is generally clearer, and the risk of complications such as intraperitoneal spread or bowel injury is reduced (33, 41).

**Figure 4:**
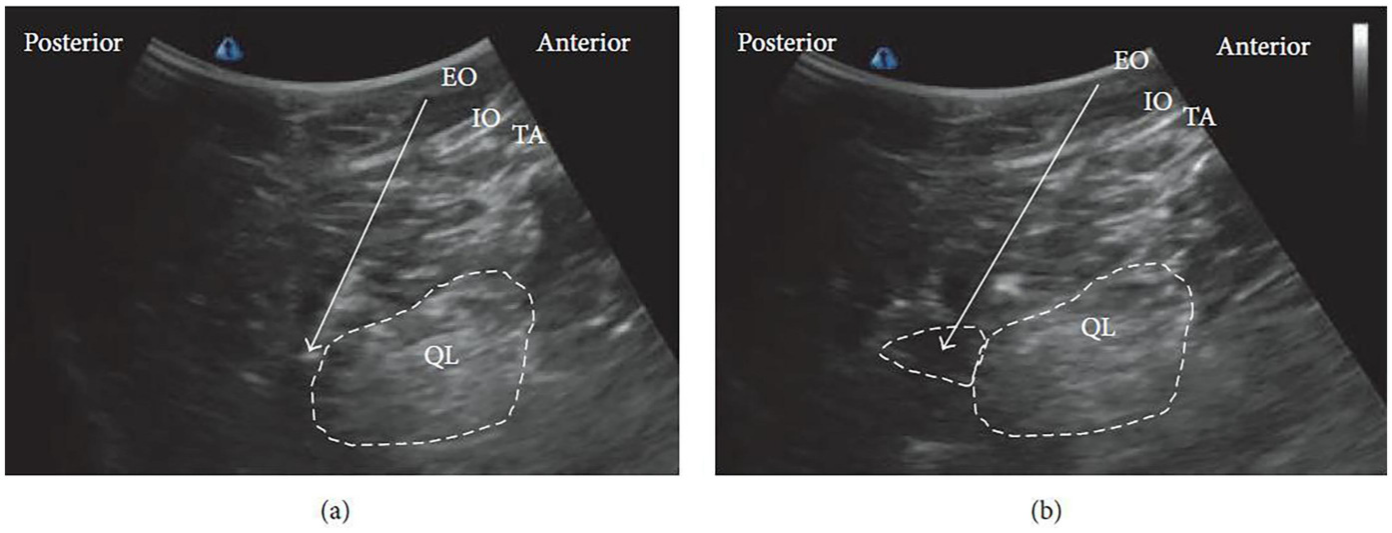
Ultrasound images of posterior QLB: **(a)** before injection and **(b)** after injection. EO, external oblique; IO, internal oblique; TA, transversus abdominis; QL, quadratus lumborum. White arrow: needle trajectory; white dotted line: spread of local anesthetic [Reproduced from Ueshima et al. (28). © John Wiley & Sons, Inc. Distributed under the Creative Commons Attribution License, CC BY 4.0].

The patient is usually positioned supine, as in the lateral QLB. In some cases, placing a pillow beneath the back can improve the working field and facilitate probe maneuverability. A low-frequency curvilinear ultrasound probe is most commonly employed. Under ultrasound guidance, the posterior margin of the QL muscle is identified, and the needle is advanced until the tip reaches this fascial plane (Figure 4a). Local anesthetic is then injected within the LIFT, situated posterior to the QL (Figure 4b).

### 4.3 Anterior quadratus lumborum block

**Figure 5:**
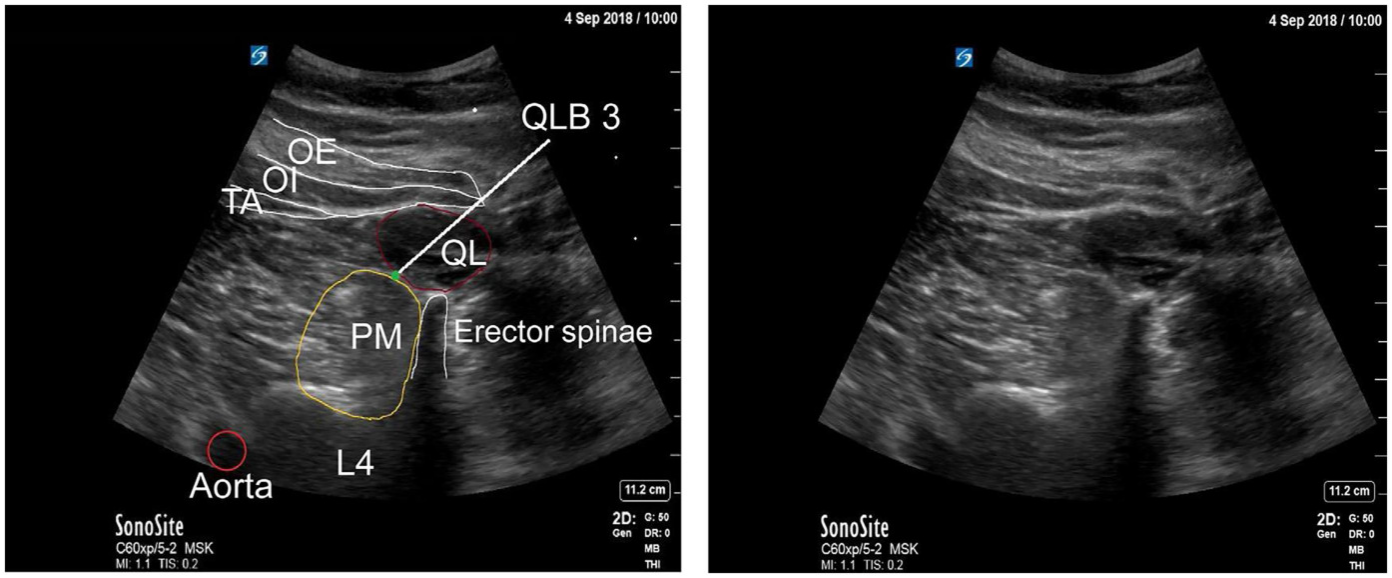
Anterior quadratus lumborum block (QLB). TA, transversus abdominis; IO, internal oblique; EO, external oblique; PM, psoas major [Reproduced from Vamnes et al. (58). © 2021 by the Croatian Medical Journal. Distributed under the Creative Commons Attribution License, CC BY 4.0].

The anterior QLB (QLB3), first described by Børglum and colleagues (42), is a transmuscular technique in which local anesthetic is deposited into the fascial plane between the anterior surface of the QL and the PM muscle (Figure 5). This anatomical space permits cranial spread of anesthetic through the diaphragmatic arcuate ligaments and the endothoracic fascia, thereby reaching the thoracic paravertebral space (39, 43). As a result, QLB3 can achieve both somatic and sympathetic blockade, depending on the extent of cranio-caudal diffusion (37, 44).

**Figure 6:**
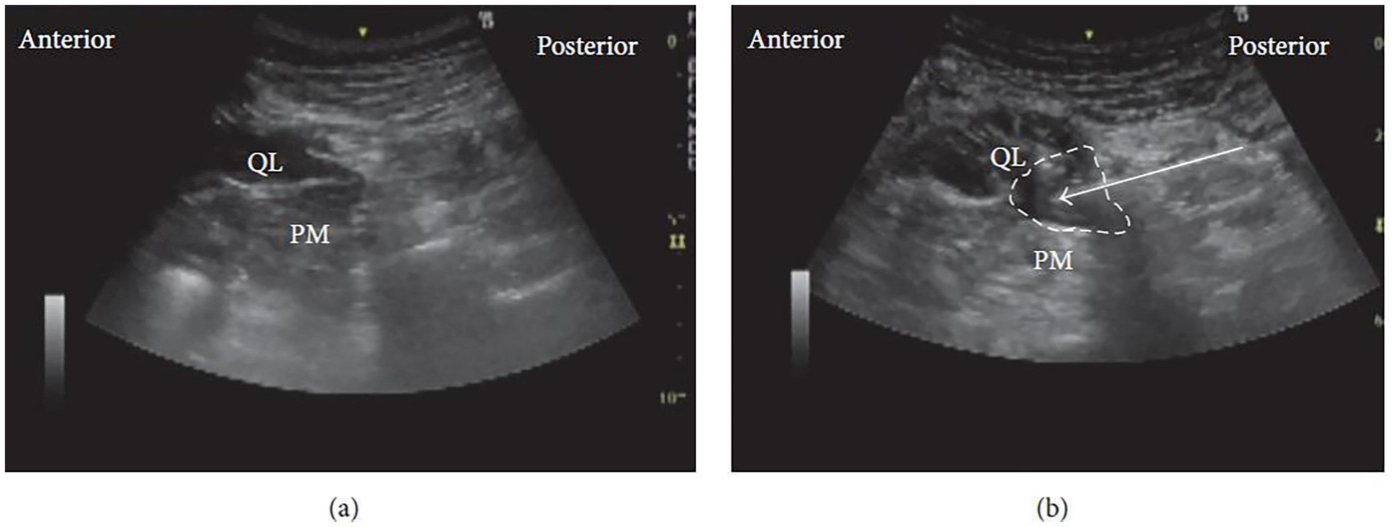
Ultrasound images of anterior QLB: **(a)** before injection and **(b)** after injection. QL, quadratus lumborum; PM, psoas muscle. White arrow: needle trajectory; white dotted line: spread of local anesthetic [Reproduced from Ueshima et al. (28). © John Wiley & Sons, Inc. Distributed under the Creative Commons Attribution License, CC BY 4.0].

In the conventional transmuscular approach, the patient is positioned laterally. A low-frequency convex probe is placed vertically above the iliac crest. The needle is inserted in-plane from the posterior margin of the probe and advanced anteromedially through the QL muscle (Figure 6a). Ultrasound visualization of the posterior vertebral and paravertebral structures is essential. Once the tip reaches the interfascial plane between the QL and PM, local anesthetic is injected, displacing the PM anteriorly (Figure 6b).

A modified technique, the subcostal anterior QLB, employs a paramedian sagittal oblique approach (33). In this method, the patient remains in the lateral decubitus position, and a convex probe is positioned approximately 3 cm lateral to the L2 spinous process. The needle is then advanced laterally into the interfascial plane between the QL and PM. Compared with the traditional approach, the PM provides a more robust protective barrier against peritoneal puncture than the relatively thin transversalis fascia.

**Figure 7:**
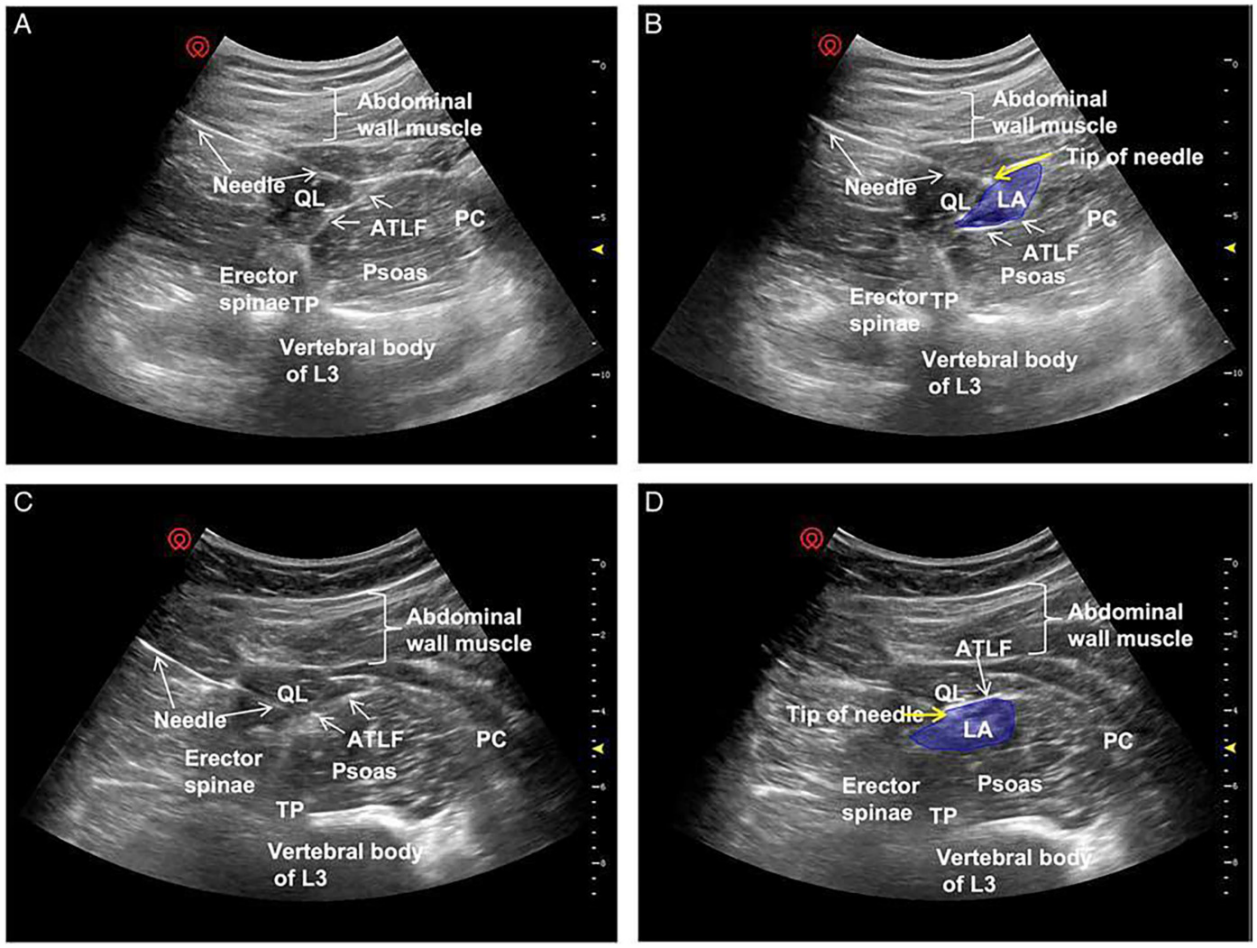
Ultrasound images of QLB3. Panels **(A,C)** show preferred ultrasound landmarks. Panel **(B)** depicts a subfascial injection of local anesthetic into the ATLF (blue), displacing the QL toward the transducer while preserving PM morphology. Panel **(D)** illustrates an extrafascial injection (blue), displacing the PM toward the vertebral body with preserved QL morphology. ATLF, anterior thoracolumbar fascia; PC, peritoneal cavity; QL, quadratus lumborum; TP, transverse process; TQL, transmuscular quadratus lumborum [Reproduced from He et al. (45). © Wolters Kluwer Health, Inc. Distributed under the Creative Commons Attribution License, CC BY 4.0].

Clinical investigations have highlighted differences in anesthetic distribution depending on whether the injection is placed subfascially or extrafascially relative to the anterior thoracolumbar fascia (ATLF) (45). He et al. (45) proposed that the ATLF serves as a barrier limiting anesthetic spread into the lumbar plexus. On ultrasound imaging, the local anesthetic appears as a spindle-shaped hypoechoic area between the ATLF and the QL. With subfascial injection, the QL shifts toward the transducer, whereas with extrafascial injection, the QL displaces the local anesthetic toward the vertebral body (Figure 7).

Subfascial injections demonstrated cranial spread through the ATLF into the endothoracic compartment, effectively blocking lower thoracic nerves, and lateral spread into the transversus abdominis plane, thus providing broader somatic coverage. Importantly, this pathway restricted direct diffusion into the lumbar plexus, thereby reducing the risk of motor weakness. By contrast, extrafascial injections tended to spread along the ATLF, sometimes crossing the 12th rib and extending into potential spaces between the ATLF and the QL. This distribution often reached the lumbar paravertebral region and the PM, increasing the likelihood of lumbar plexus involvement and limb weakness (46, 47).

Patterns of sensory blockade also differed: subfascial injections typically provided coverage from T7–T8 to T12–L1, whereas extrafascial injections extended from T11–T12 to L3–L4 (45). Taken together, these findings suggest that subfascial anterior QLB offers more consistent analgesia while reducing the incidence of undesired motor effects.

### 4.4 Intramuscular quadratus lumborum block

The intramuscular QLB (QLB4) is performed by depositing local anesthetic directly into the belly of the QL muscle (48). Owing to this injection site, it is commonly referred to as the intramuscular approach (37).

**Figure 8:**
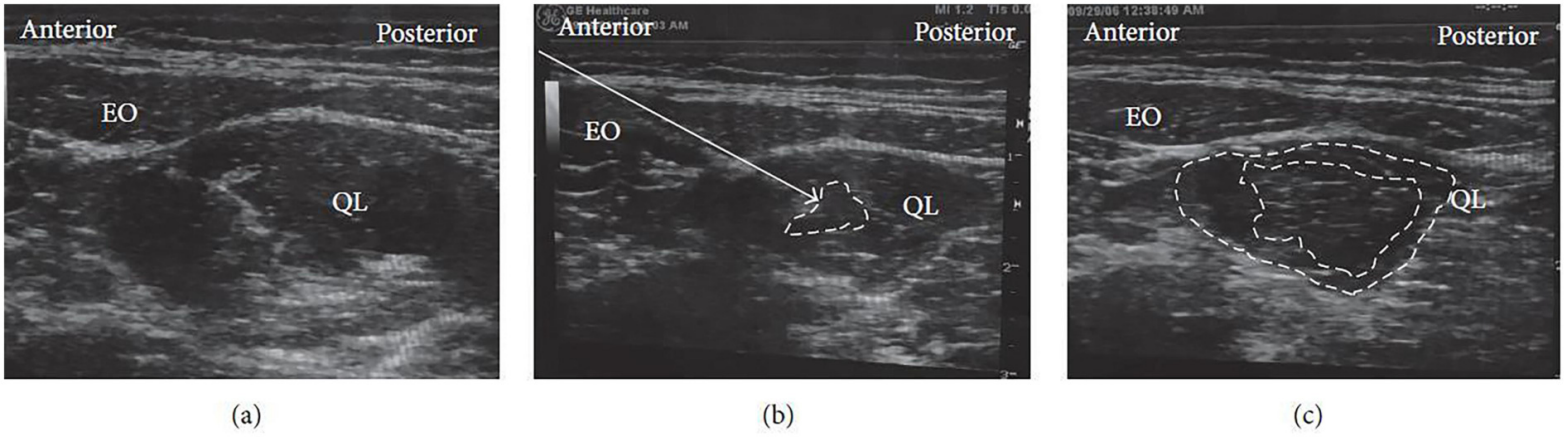
Ultrasound images of intramuscular QLB: **(a)** before injection, **(b)** during test injection, and **(c)** after injection. EO, external oblique; QL, quadratus lumborum. White arrow: needle trajectory; white dotted line: spread of local anesthetic within (b) or between (c) muscle layers [Reproduced from Ueshima et al. (28). © John Wiley & Sons, Inc. Distributed under the Creative Commons Attribution License, CC BY 4.0].

For this technique, the patient is generally positioned supine, as in the lateral QLB. A high-frequency linear ultrasound probe is placed just above the iliac crest. Under real-time ultrasound guidance, the needle is advanced until it penetrates the fascial layer and enters the substance of the QL muscle (Figure 8a). A small test injection is administered to confirm correct placement, with successful delivery indicated by visible dispersion of the injectate among the muscle fibers (Figure 8b). Effective block performance is further characterized by diffusion of the anesthetic within the QL itself or between the muscle and its adjacent fascial layers (Figure 8c) (28).

## 5 Choice of anesthetic

Currently, no universally accepted guideline exists regarding the most effective local anesthetic agent, its optimal concentration, or the ideal volume for QLB administration (36). Among the available agents, ropivacaine is most frequently used because of its favorable pharmacological profile and reduced cardiotoxicity and neurotoxicity compared with bupivacaine. Typical dosing regimens include 0.2–0.4 ml/kg of 0.2%–0.5% ropivacaine or 0.1%–0.25% bupivacaine per side (37). For bilateral blocks, the total dosage must be carefully adjusted to avoid systemic toxicity, with 150 mg of ropivacaine generally considered the safe upper limit (48). A notable advantage of QLB is the prolonged duration of analgesia, which often exceeds 24 h, making it particularly valuable for extended postoperative pain control (48).

## 6 Mechanisms of quadratus lumborum block

The QLB is unique in its ability to provide both somatic and visceral analgesia. Nonetheless, the extent of sensory coverage is variable, with most reports describing dermatomal spread between T7 and L1 (28, 32, 42, 49, 50). Some studies have documented cranial extension as high as T4–T5 (51), while occasional caudal diffusion has been observed down to L2–L3 (52).

Cadaveric dissections and clinical investigations have helped delineate the characteristic spread patterns associated with each QLB approach:

- QLB1 (lateral): most commonly produces sensory blockade between T7 and L1, with injectate predominantly diffusing into the transversus abdominis plane (52).
- QLB2 (posterior): generally, covers T7–L1, with local anesthetic spreading within the middle layer of the thoracolumbar fascia (TLF) (52).
- QLB3 (anterior/transmuscular): typically achieves wider coverage, ranging from T7 to L2, and may extend into both the lumbar and thoracic paravertebral spaces (53).
- QLB4 (intramuscular): usually provides a more limited distribution, often confined to T7–T12 (48).

A comparative overview of these techniques, including their typical dermatomal coverage and characteristic spread patterns, is presented in Table 1.

**Table 1:** Anatomical approaches to Quadratus lumborum block and dermatomal coverage.

Type	Injection site	Spread	Typical dermatomes	Advantages	Limitations
QLB1 (Lateral)	Anterolateral to QL, near transversalis fascia	Cranial spread possible	T7–L1	Simple, ultrasound-guided	Less reliable visceral block
QLB2 (Posterior)	Posterior to QL (LIFT)	Good fascial spread	T7–L1	Reliable, safe	May require larger volume
QLB3 (Anterior/Transmuscular)	Between QL and psoas major	Can reach paravertebral space	T7–L2	Strong analgesia, visceral coverage	Risk of motor weakness
QLB4 (Intramuscular)	Within QL belly	Limited	T7–T12	Technically simple	Shorter duration

## 7 Results of quadratus lumborum block in laparoscopic cholecystectomy

**Figure 9:**
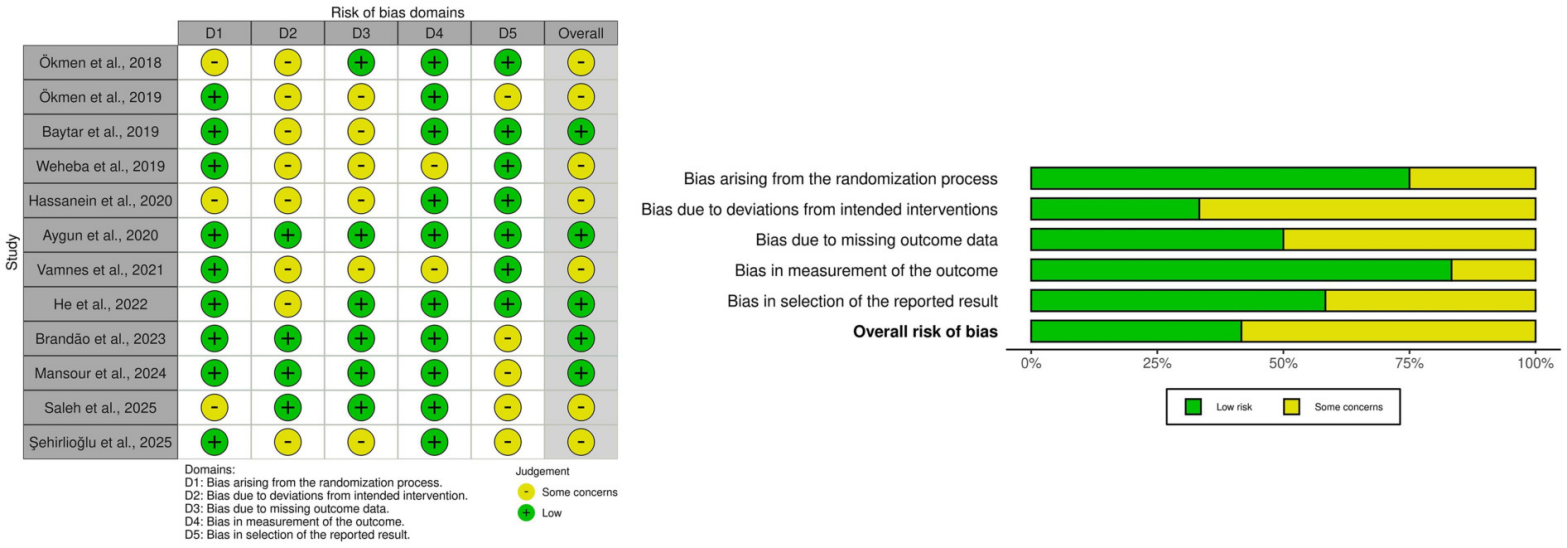
Risk of bias (RoB 2) summary: per-study domain ratings (left) and domain-wise distribution across studies (right).

Despite the routine implementation of multimodal analgesia in LC, the challenge of achieving consistently adequate postoperative pain control persists. This has stimulated growing interest in regional anesthesia techniques, including the QLB. Although the body of literature remains relatively limited, several randomized controlled trials (RCTs) and one retrospective study have provided important insights into the efficacy, limitations, and comparative performance of different QLB approaches in this setting (Table 2). Below, the most relevant studies are summarized and critically analyzed. We summarize RoB 2 findings graphically in Figure 9. Importantly, the sole retrospective comparative study (54) was judged high overall risk of bias by ROBINS-I, driven principally by serious confounding and selection bias, with additional concerns in intervention classification and outcome measurement; accordingly, we treat its results as hypothesis-generating only and do not draw causal inferences from it. Because of clinical and methodological heterogeneity and the predominance of RoB 2 “some concerns” among trials (notably in deviations from intended interventions and selective reporting), we prespecified qualitative, direction-of-effect synthesis rather than quantitative pooling; when head-to-head trials conflicted, we prioritized consistency of direction, rescue-analgesia behavior, and time-course over isolated *p*-values. These methodological choices and risk-of-bias findings directly inform the interpretation of results and the wording of conclusions: the manuscript's overall statements have been moderated to reflect between-study heterogeneity, imprecision, and domain-level limitations, emphasizing that while QLB can be a reasonable component of multimodal analgesia for LC—especially for early pain and opioid sparing—head-to-head evidence frequently shows analgesic equivalence to TAPB or ESPB, and confidence is tempered by design constraints and incomplete protocol standardization across studies.

**Table 2:** Trials on Quadratus lumborum block for laparoscopic cholecystectomy.

Author, Year	Study design & Patients	QLB Technique	Control/Comparison	Main outcomes	Key findings
Kulhari et al. (54)	Retrospective, *n* = 19	Posterior QLB	Systemic analgesia only	VAS at 3, 12, 24 h; chronic pain incidence	Lower pain scores with QLB; none developed chronic pain vs. 33% in control
Ökmen et al. (55)	RCT, *n* = 60	Posterior QLB	Saline placebo	VAS at rest and movement, tramadol consumption	LB significantly reduced pain and tramadol use at all time points
Ökmen et al. (56)	RCT, *n* = 60	Posterior vs. Lateral QLB	Both groups with tramadol PCIA	Pain scores, tramadol use, side effects	No significant difference between posterior and lateral QLB; both effective
Weheba et al. (25)	RCT, *n* = 98	Posterior QLB	Subcostal TAPB	VAS at 1, 6, 12, 24 h; fentanyl consumption; time to first rescue analgesia	Similar pain scores; fewer patients required opioids in QLB group; longer time to rescue analgesia
Hassanein et al. (57)	RCT, *n* = 90	Posterior QLB	ESPB; no block	VAS, time to first analgesia, fentanyl use	Both ESPB and QLB reduced pain vs. control; ESP slightly superior in duration, but QLB effective and opioid-sparing
Aygun et al. (22)	RCT, *n* = 80	Posterior QLB (bilaterally)	ESPB; both with general anesthesia + multimodal analgesia	Opioid consumption at 24 h; NRS pain scores	No significant difference between QLB-II and ESPB; both improved analgesia compared with baseline
Vamnes et al. (58)	RCT, *n* = 70	Anterior QLB (bilaterally)	Placebo (saline) and standard multimodal analgesia	Opioid use, pain scores (NRS), PONV up to 48 h	No difference in opioid use or pain; QLB group had reduced PONV
Baytar et al. (59)	RCT, *n* = 120	Posterior QLB	Subcostal TAPB	Pain, time to first analgesia, opioid use	No significant difference; TAPB simpler technically, but QLB prolonged analgesia in some cases
He et al. (45)	RCT, *n* = 60	Anterior QLB, subfascial vs. extrafascial	Two injection planes	Dermatomal spread, VAS, rescue analgesia	Subfascial QLB provided broader coverage, lower pain, and fewer motor side effects than extrafascial
Brandão et al. (24)	RCT, *n* = 51 (same cohort as below)	Anterior QLB (bilaterally)	General anesthesia + venous analgesia	VAS at 1, 4, 24 h; IL-6, CRP, cortisol; respiratory pressures	QLB reduced pain at 24 h, attenuated IL-6 and cortisol rise, improved early respiratory pressures
Brandão et al. (26)	RCT, *n* = 51 (same cohort as above)	Anterior QLB (bilaterally)	General anesthesia + venous analgesia	VAS at 1, 4, 24 h; opioid use; IL-6, CRP, cortisol; lung function	QLB reduced pain at all timepoints, decreased cortisol at 4 h, improved MIP/MEP recovery, minor effect on CRP
Mansour et al. (23)	RCT, *n* = 90	Transmuscular QLB (± dexamethasone)	Saline placebo; bupivacaine alone	VAS, analgesia duration, opioid use, satisfaction	Dexamethasone prolonged analgesia and reduced opioid need vs. bupivacaine alone
Saleh et al. (60)	RCT, *n* = 70	Lateral QLB	Intraperitoneal + port infiltration	VAS, opioid use, time to first analgesia	QLB reduced early pain, delayed rescue analgesia, and decreased opioid use
Şehirlioğlu et al. (61)	RCT, *n* = 108	Anterior QLB	Modified TAPA block	NRS, opioid use, dermatomal spread	QLB and m-TAPA provided similar analgesia; QLB mainly T10–L1, m-TAPA T7–T10

### 7.1 Posterior quadratus lumborum block

One of the most illustrative comparative studies was conducted by Hassanein et al. (57), who randomized patients into three groups: bilateral ESPB, bilateral posterior QLB, or no block. Both regional techniques were performed under ultrasound guidance using 20 ml of 0.25% bupivacaine per side. Surgery commenced 15 min after block administration, and postoperative analgesia consisted of ketorolac (30 mg). Pain was assessed every 8 h using the Visual Analog Scale (VAS), with fentanyl (1 µg/kg) administered when VAS exceeded 3. Both regional techniques significantly improved analgesia compared with controls. The time to first request for rescue analgesia was longest in the ESPB group (13.5 ± 4.5 h), followed by the QLB group (8.7 ± 4.1 h), and shortest in controls (1.3 ± 0.4 h; *p* < 0.001). Similarly, the total duration of analgesia was greater in the ESPB (11.2 ± 2 h) and QLB (10.0 ± 3.4 h) groups than in controls (1.3 ± 0.4 h). Fentanyl requirements mirrored these findings: they were highest in controls (98.9 ± 34.1 µg/kg) and significantly lower in ESPB (79.5 ± 21.2 µg/kg) and QLB (83 ± 19.6 µg/kg) groups (*p* = 0.04). Importantly, VAS scores during coughing were markedly lower in ESPB and QLB groups compared with controls at 1 and 2 h postoperatively. At later intervals (8 and 16 h), ESPB produced slightly lower pain scores than QLB, suggesting modest superiority in sustaining analgesia. Overall, this study confirmed that both ESP and posterior QLB provide effective analgesia and opioid-sparing effects after LC, with ESPB offering marginally longer relief.

Kulhari et al. (54) directly compared posterior QLB with standard systemic analgesia. Static and dynamic VAS scores were recorded immediately postoperatively and at 3, 12, and 24 h. Patients receiving posterior QLB consistently reported significantly lower pain scores at all time points (*p* < 0.05). Notably, none of the QLB patients developed chronic postoperative pain, whereas 33.3% of patients in the control group did, suggesting a potential long-term protective effect against chronic pain. Given the retrospective design, small sample size, and lack of blinding, these findings should be regarded strictly as preliminary and hypothesis-generating and do not justify claims that QLB prevents chronic pain. Confirmation will require adequately powered, blinded randomized trials with standardized assessment of chronic pain at ≥3–6 months.

In another RCT, Ökmen et al. (55) enrolled 60 patients undergoing LC and randomized them to receive either posterior QLB with 0.3 ml/kg of 0.25% bupivacaine in addition to tramadol-based patient-controlled intravenous analgesia (PCIA) (group B) or saline instead of bupivacaine (group S). Pain scores were assessed at rest (30 min, 2, 6, 12, 24 h) and during movement (2, 6, 12, 24 h). Tramadol consumption was recorded at 6, 12, and 24 h. Patients in the bupivacaine group consistently reported significantly lower pain scores at all time points (*p* < 0.001), both at rest and during movement. Mean tramadol consumption was also markedly reduced compared with the saline group. This study provided strong evidence that posterior QLB with bupivacaine is clinically effective and reduces opioid requirements as part of multimodal analgesia in LC.

In a subsequent RCT, Ökmen et al. (56) compared posterior and lateral QLB in 60 patients undergoing elective LC, with tramadol PCIA used in both groups. Outcomes included cumulative tramadol consumption, VAS scores at rest and during movement, and incidence of side effects. No statistically significant differences were observed between groups. Both posterior and lateral QLB were equally effective, indicating that the choice of technique may depend more on operator preference, ultrasound visibility, and patient anatomy than on differences in analgesic efficacy.

Aygun et al. (22) compared QLB type II with ESPB in 80 patients and reported no significant differences in opioid consumption or pain scores between groups. Both techniques provided satisfactory analgesia and effectively reduced postoperative pain compared with baseline.

Baytar et al. (59) randomized 120 patients to bilateral subcostal TAPB or bilateral posterior QLB, each using 0.3 ml/kg bupivacaine. Primary outcomes included pain intensity, time to first analgesic request, total analgesic consumption, and complication rates. No significant differences were observed between groups. However, TAPB was noted to be technically simpler and faster to perform, potentially making it more advantageous in routine clinical practice.

By contrast, another RCT (25) involving 106 patients compared posterior QLB with subcostal TAPB. Although total fentanyl consumption did not differ significantly, fewer patients in the QLB group required opioids (17/48 vs. 28/50). Additionally, the time to first rescue analgesia was significantly longer in the QLB group, suggesting a more sustained analgesic effect. Pain scores and incidence of postoperative nausea and vomiting (PONV) did not differ significantly between groups.

Taken together, these findings suggest that while TAPB and QLB provide comparable analgesia in some trials, posterior QLB may have an advantage in prolonging the duration of pain relief and reducing the proportion of patients requiring opioids. This positions posterior QLB as a potentially valuable technique in opioid-sparing strategies for LC.

### 7.2 Anterior (transmuscular) quadratus lumborum block

Several studies have investigated the anterior quadratus lumborum block (QLB3), though results remain heterogeneous.

A randomized controlled trial (58) compared anterior QLB with ropivacaine, placebo QLB with saline, and standard intravenous/oral analgesia. No significant differences were observed in opioid consumption or pain scores at 1, 2, 24, and 48 h. However, the incidence of PONV was lower in the ropivacaine group, suggesting ancillary benefits beyond analgesia. The authors concluded that anterior QLB should not yet be adopted as a routine technique for LC.

Another trial (23) assessed the impact of adding dexamethasone to ropivacaine in anterior QLB. Ninety patients were randomized into three groups: control (saline), bupivacaine, and bupivacaine with dexamethasone. The time to first rescue analgesia was longest in the dexamethasone group (18 h), compared with bupivacaine alone (14 h) and saline (0.8 h). Both bupivacaine groups demonstrated superior analgesia relative to saline, but the addition of dexamethasone clearly prolonged block duration. Patient satisfaction scores were consistent with these findings, supporting the use of dexamethasone as an adjuvant to enhance block efficacy.

He et al. (45) specifically compared subfascial and extrafascial anterior QLB using 30 ml of 0.33% ropivacaine. Subfascial injections achieved broader dermatomal coverage (T7–L1 vs. T11–L3 with extrafascial), significantly lower VAS scores at subxiphoid and right subcostal port sites during the first 36 h, and reduced requirements for rescue opioids and parecoxib. By contrast, extrafascial injections more frequently spread to the lumbar plexus, resulting in a higher incidence of lower-limb motor weakness.

Two publications by Brandão et al. (24, 26), derived from the same randomized clinical trial involving 51 LC patients, evaluated the addition of anterior QLB to standard anesthesia. The block significantly reduced postoperative pain intensity, decreased opioid consumption, and facilitated earlier recovery of respiratory muscle function. Moreover, it modulated systemic inflammatory and neuroendocrine responses: patients receiving the block exhibited a delayed rise in interleukin-6 and lower cortisol levels in the early postoperative period, while C-reactive protein levels showed less pronounced changes. These findings suggest that anterior QLB may provide dual benefits—effective analgesia alongside partial attenuation of the inflammatory and stress response to surgery.

A prospective, randomized, single-blinded trial compared anterior QLB with modified thoracoabdominal nerve block through the perichondrial approach (m-TAPA) in 108 patients undergoing LC (61). Patients received either bilateral anterior QLB (*n* = 55) or bilateral m-TAPA (*n* = 53). Postoperative outcomes within the first 24 h included analgesic consumption, Numeric Rating Scale (*N*RS) scores at rest and movement, time to first rescue analgesic, and side effects. No significant differences were observed between groups in opioid consumption, NRS scores, intraoperative remifentanil use, or complication rates. Dermatomal involvement differed: m-TAPA primarily covered T6–T10 dermatomes, whereas anterior QLB predominantly covered T10–L1. Both techniques provided effective and comparable analgesia in the first 24 h, reinforcing their role in multimodal, opioid-sparing strategies for LC.

Overall, anterior QLB demonstrates potential as an effective component of perioperative analgesia, but its efficacy depends heavily on accurate injection relative to the thoracolumbar fascia. The subfascial approach appears superior to extrafascial placement in terms of both coverage and safety, while the addition of dexamethasone reliably prolongs block duration.

### 7.3 Lateral quadratus lumborum block

A randomized double-blind study compared the efficacy of ultrasound-guided bilateral lateral QLB with intraperitoneal and periportal bupivacaine infiltration for postoperative pain management following LC (60). Seventy patients (aged 21–60 years) were equally allocated into two groups. Patients in the QLB group demonstrated significantly lower pain scores during the first 6 h after surgery and required less opioid and overall analgesic consumption within 24 h compared with the infiltration group. Furthermore, the time to first rescue analgesia was markedly prolonged in the QLB group, underscoring the clinical utility of this approach.

### 7.4 Discussion

Taken together, the evidence on QLB after LC is heterogeneous. Several RCTs demonstrate clinically meaningful reductions in pain and/or opioid consumption vs. placebo or no block, yet multiple head-to-head comparisons with other regional techniques—most commonly the TAPB or ESPB—show no significant differences in primary outcomes, suggesting analgesic equivalence when embedded in robust multimodal regimens. The direction and magnitude of effect appear sensitive to differences in QLB approach (posterior/lateral vs. anterior), injectate volume and concentration, adjuvant use (e.g., perineural dexamethasone), comparator selection, and the timing and definition of pain assessments. Although comparisons with TAP remain inconclusive overall, posterior QLB may offer advantages in prolonging analgesia and reducing opioid consumption in selected contexts, whereas the evidence base for anterior QLB is less mature; encouraging signals—particularly with subfascial injection and dexamethasone—do not yet justify routine adoption without further high-quality confirmation.

A balanced appraisal underscores that not all trials favor QLB for LC. Neutral or negative findings in primary outcomes have been reported, including an anterior QLB RCT that showed no reduction in opioid use or pain scores vs. placebo within a robust multimodal protocol, despite lower postoperative nausea and vomiting (PONV) in the active-block arm (58). Head-to-head trials frequently report equivalence: posterior QLB vs. ESPB (22) and posterior QLB vs. subcostal TAPB (59) yielded no significant differences in pain intensity or opioid consumption under standardized co-analgesia. In another randomized comparison, ESPB achieved a modestly longer duration of analgesia than posterior QLB, though both were superior to no block (57). Conversely, lateral QLB outperformed intraperitoneal plus periportal infiltration for early pain and time to first rescue in one trial (60), emphasizing how comparator choice can amplify or attenuate apparent effect sizes. Collectively, these observations position QLB as a reasonable component of opioid-sparing multimodal care, while superiority over TAP, ESP, or m-TAPA is inconsistent across studies (22, 57, 59, 61).

Between-study heterogeneity likely drives much of the variability in effect estimates. 1. *Technique and injection plane*. Outcomes differ across QLB1 (lateral), QLB2 (posterior), QLB3 (anterior/transmuscular), and QLB4 (intramuscular). Subfascial vs. extrafascial placement in anterior QLB meaningfully alters dermatomal coverage, sympathetic involvement, and motor effects; subfascial injection has been associated with broader coverage, lower pain, and fewer motor symptoms than extrafascial (45). Posterior and lateral approaches often provide similar clinical analgesia (56) and may be simpler or more motor-sparing in routine practice. 2. *Local anesthetic and laterality*. Regimens vary in drug (ropivacaine vs. bupivacaine), concentration, and volume (commonly 20–30 ml per side and frequently bilateral), with implications for cranio-caudal spread and duration; the lack of dose-finding trials leaves the optimal schema uncertain (36, 48). 3. *Adjuvants*. Perineural dexamethasone can prolong block duration and reduce opioid requirements, but its use and dosing are inconsistent between and within trials (23). 4. *Comparators and co-interventions*. Controls span saline sham, active regional techniques (subcostal TAPB, ESPB, m-TAPA), and surgeon infiltration; systemic regimens range from patient-controlled opioid analgesia to scheduled NSAIDs/acetaminophen with or without routine IV dexamethasone, potentially diluting or magnifying QLB's incremental benefit (22, 57–61). 5. *Outcome frameworks*. Pain scales (VAS vs. NRS), testing at rest vs. movement, and assessment windows (from 30 min to 48 h) are not standardized; conversion of opioids to morphine equivalents is inconsistently reported. Patient-centered outcomes (e.g., QoR-15, readiness for discharge, functional recovery) and longer-term pain are infrequently captured, limiting inference about broader recovery trajectories.

Methodological constraints further temper certainty. Many trials are single-center and small, raising the risk of type II error when equivalence is concluded; blinding is inherently imperfect around block performance, and deviations from intended interventions or selective reporting contribute to RoB 2 “some concerns” in multiple domains. Block success/failure is not uniformly verified with sensory mapping; operator experience is seldom quantified; and adverse events such as transient motor weakness with anterior/extrafascial spread may be under-ascertained. The sole comparative retrospective analysis suggesting reduced chronic pain with QLB (54) is highly confounded (selection, indication), so any implication that QLB prevents chronic postoperative pain must be viewed as hypothesis-generating rather than causal. Signals such as “fewer patients required any opioid” despite similar total doses warrant cautious interpretation given multiplicity, potential type-I error, and baseline imbalances (25). In some RCTs, anterior QLB was also associated with reduced pain-related stress responses (lower cortisol/IL-6) and improved early respiratory function (24, 26), but these findings arise from limited datasets that require replication.

*Practical interpretation*. Within contemporary multimodal LC pathways, QLB commonly improves early pain and reduces opioid exposure, but equivalence to TAPB or ESPB is frequent. If QLB is selected, technique should align with the analgesic target and the operator's expertise: posterior or lateral QLB for reliable somatic coverage with procedural simplicity; anterior QLB when broader visceral coverage is prioritized—preferably using subfascial placement to mitigate motor effects—and considering perineural dexamethasone as an adjunct (23, 45, 56) (Box 1).

Box 1 **Take-Home: Algorithm to Guide QLB Technique Selection**

1. Screen. Contraindications to deep/neuraxial-adjacent blocks? If yes → superficial options (TAPB ± port-site infiltration). If no → Step 2.
2. Dominant pain source. Somatic/ports, day-case → QLB1 or QLB2. Visceral/diaphragmatic expected → QLB3 (anterior/transmuscular).
3. Operator & window. Comfortable with transmuscular and good sono-window → QLB3 (target subfascial plane between QL and psoas). Limited experience/poor view → QLB2 (posterior/LIFT).
4. Practical tips. Bilateral without turning → QLB1/QLB2 (supine). High BMI/landmarking issues → QLB2 (curvilinear). Early ambulation/motor-sparing → avoid QLB3 extrafascial; prefer QLB2/QLB1 or QLB3 subfascial. Strong multimodal systemic analgesia and only port pain → QLB1/QLB2 usually sufficient.

## 8 Clinical implications and future directions

Current evidence indicates that the QLB is a valuable component of multimodal analgesia for LC. Both posterior and lateral approaches have consistently demonstrated reduced postoperative pain scores, prolonged time to first rescue analgesia, and significant opioid-sparing effects. These benefits are particularly relevant in the contemporary clinical landscape, where minimizing perioperative opioid exposure has become both a medical and societal priority. Moreover, the potential of QLB to attenuate the surgical inflammatory response merits further investigation; any effect on the transition from acute to chronic pain remains unproven and is, at best, hypothesis-generating based on limited, low-quality data.

Despite these encouraging findings, several limitations in the current literature must be acknowledged. Most randomized controlled trials are single-center investigations with relatively small sample sizes, and significant heterogeneity persists in block techniques, volumes and concentrations of local anesthetic, and use of adjuvants. Comparative studies against other regional techniques, such as TAP and ESP blocks, remain inconclusive, while the clinical utility of anterior and intramuscular QLB approaches has yet to be firmly established.

Beyond differences in analgesic outcomes, the choice between TAPB, ESPB, and QLB should weigh workflow and safety considerations. TAPB is typically the most familiar and fastest to execute with consistent superficial sono-anatomy, but it predominantly targets somatic pain and often provides a shorter duration of effect. ESPB is technically forgiving with a straightforward ultrasound window and broad cranio-caudal spread, yet visceral coverage can be variable. QLB offers deeper, potentially longer-lasting somatic–visceral analgesia when correctly sited; posterior/lateral approaches are generally simpler and motor-sparing, whereas the anterior (transmuscular) approach demands greater expertise and meticulous subfascial needle placement to minimize lumbar plexus spread. Within multimodal pathways, technique selection should reflect operator experience, ultrasound view, anticipated dominant pain source (port-site vs. visceral/diaphragmatic), positioning constraints, and time available in a day-case setting; adjuvants (e.g., perineural dexamethasone) may further prolong duration.

Future research should prioritize large-scale, multicenter, randomized controlled trials with standardized protocols, longer follow-up periods, and patient-centered outcomes. Such studies will be essential to establish the comparative efficacy, safety, and long-term benefits of QLB, and to clarify its optimal role within multimodal analgesic strategies for LC.

## 9 Conclusion

Despite the routine use of systemic analgesics, patients undergoing laparoscopic cholecystectomy frequently experience significant postoperative pain. Furthermore, systemic analgesia is often accompanied by adverse effects such as postoperative nausea and vomiting, which further compromise patient comfort and recovery. QLB is a useful option within multimodal analgesia for LC, with several trials showing reduced early pain and opioid use; however, comparative efficacy vs. TAPB or ESPB is often similar, and not all studies demonstrate superiority. Claims that QLB prevents chronic postoperative pain after laparoscopic cholecystectomy are not supported by high-quality evidence. Until adequately powered, blinded RCTs with long-term follow-up are available, such statements should be avoided or explicitly labeled as preliminary.

Given the heterogeneity of techniques, dosing, comparators, and moderate risk-of-bias across studies, our overall conclusions are moderated. Larger, standardized RCTs with robust blinding, predefined primary endpoints (pain at rest/movement), and transparent co-analgesic protocols are needed to define the comparative and approach-specific benefits of QLB.
